# Perceptions of Community Members and Healthcare Workers on Male Involvement in Prevention of Mother-To-Child Transmission Services in Khayelitsha, Cape Town, South Africa

**DOI:** 10.1371/journal.pone.0133239

**Published:** 2015-07-28

**Authors:** Alice Norah Ladur, Christopher J. Colvin, Kathryn Stinson

**Affiliations:** 1 Centre for Infectious Disease Epidemiology and Research (CIDER), University of Cape Town, Cape Town, South Africa; 2 Department of Community Health, Africa Renewal University, Kampala, Uganda; UCL Institute of Child Health, University College London, UNITED KINGDOM

## Abstract

Involving male partners of pregnant women accessing PMTCT programs has the potential to improve health outcomes for women and children. This study explored community members’ (men and women) and healthcare workers’ perceptions of male involvement in the prevention of mother-to-child transmission of HIV in Khayelitsha, South Africa. Two focus group discussions were held with 25 men of unknown HIV status and one focus group discussion held with 12 HIV-positive women in the community. In depth interviews were conducted with four HIV-positive couples and five service providers purposely sampled from the community and a health facility, respectively. Both men and women interviewed in this study were receptive towards male involvement in PMTCT. However, men were reluctant to engage with health services due to stigma and negative attitudes from nurses. This study also found HIV testing, disclosure and direct health worker engagement with men increases male involvement in PMTCT. Using men in the media and community to reach out to fellow men with prevention messages tailored to suit specific audiences may reduce perceptions of antenatal care as being a woman`s domain.

## Introduction

In South Africa, an estimated 5.6 million people were living with HIV in 2011, while AIDS-related illnesses accounted for 35% of all deaths in children less than five years of age [1, 2].

Prevention of mother-to-child transmission (PMTCT) offers a unique opportunity to reduce the number of HIV-related deaths in children under five years as well as serve as an entry point to HIV care for mothers and their families, promoting maternal and family health [3]. Similar to other African countries, South Africa`s PMTCT programme focuses on; voluntary HIV testing and counselling, antiretroviral treatment or prophylaxis for the mother and infant, modified obstetric practices, and modified infant–feeding counselling [4,5]. HIV services are integrated within primary health care, TB, maternal, new born and child health enhancing a family centred approach [6]. The South African PMTCT guidelines were revised in 2010 and are currently being implemented in almost all (98%) the health facilities in South Africa [7]. Voluntary HIV testing is provided to all pregnant mothers at first antenatal care (ANC) visit, all HIV positive women who are pregnant, breast feeding or within one year post-partum are initiated on lifelong ART irrespective of CD4 cell count. Exclusive breast feeding is recommended for infants during the first six months [6].

HIV prevalence among women accessing antenatal services is still high at 29.5% with 280,000 pregnant women requiring treatment for prevention of mother-to-child transmission of HIV in 2012 [8]. Despite the progress made in improving PMTCT coverage in South Africa, a considerable number of women report late for the first antenatal care visit, are initiated on ART late and are loss to follow up upon delivery [9,10,11]. Clouse et al, 2013 reported a median gestational age at HIV testing of 26 weeks and a high rate of loss to follow up at 57% suggesting missed opportunities in the programme

It is, therefore, important to explore different ways in which PMTCT programmes could be enhanced in order to improve outcomes for women and men accessing PMTCT services in South Africa.

Considerable attention has been drawn to the potential influence of male involvement in sexual and reproductive health in Africa [12, 13]. Male involvement has the potential to bring about change because of the social power men hold in patriarchal settings. According to Theuring et al. (2009), couple testing may serve as an entry point to women utilising PMTCT services in patriarchal communities and in settings where women have limited autonomy in decision-making [14]. Men’s role in providing emotional and material support to partners including financial support, permission to access health care, monitoring drug compliance and helping out on household chores have been identified in several studies as ways to improve women’s uptake of PMTCT services [15,16,17].

However, low levels of male HIV testing (even lower in ANC) have been reported in several studies conducted in various parts of Africa despite scale up of the provision of voluntary counselling and testing (VCT) services and educational programmes. Studies conducted in Cameroon, Ivory Coast, Kenya, Tanzania, Malawi, South Africa and Burkina Faso reported low uptake of HIV testing services by men citing a number of reasons such as; fear of knowing one`s seropositive status, time constraints, fear of stigma and using the woman’s HIV status as a criteria confirming their own status (referred to as ‘proxy testing’) [14, 18]. Lack of partner support and men’s limited knowledge about the link between antenatal care and prevention of HIV, for example, often acts as a hindrance to seeking early and effective care [19, 20]. Low levels of active male participation in antenatal care and PMTCT have been reported in South Africa [20, 21]. Although there is increasing evidence that men can make a difference in maternal health, little is known about under what conditions men choose to get involved. Furthermore, little is known about the factors that influence male participation in PMTCT programmes in South Africa, from either client or services’ perspectives. Without an understanding of the barriers and enablers to male participation in PMTCT, it will be difficult to implement PMTCT services effectively. The male partner plays a role in terms of lowering a woman`s risk of acquiring HIV, prevention of unplanned pregnancies, joint decision making on HIV testing, ART treatment and infant feeding method [22,23,24]. This study aimed to explore community members’ (men and women) and healthcare workers’ perceptions of male involvement in PMTCT in order to provide insights into possible ways of increasing male partner participation in antenatal care and improve outcomes of pregnant women accessing PMTCT services.

## Methods

The study was conducted in the Khayelitsha sub-district of the Western Cape Province in South Africa. Khayelitsha is representative of many poor urban areas in South Africa, with a large proportion of the population having migrated from rural areas. The population is estimated at 500,000, with the majority of inhabitants living in informal dwellings [2, 25]. Poverty, unemployment and crime as well as sexual violence in Khayelitsha are rife. Khayelitsha has a considerably high antenatal HIV prevalence estimated at 26% in 2010 [26].

Data for this study was collected using exploratory qualitative methods: focus group discussions and in-depth interviews between July and October 2010. The inclusion criteria consisted of men and women aged 18–40 years living in Khayelitsha. Men of unknown HIV status and HIV-positive women who were accessing PMTCT services at the time of the study or who had accessed/utilised the PMTCT service in the last 2 years prior to the study were eligible to participate. We did not ask if any of their children were HIV positive. Key informants involved in PMTCT service provision such as doctors, mid-wives, counsellors, non–governmental organisations and community health workers were also recruited to participate in the study.

Two focus group discussions were held with 25 men of unknown HIV status aged between 18–40 years living in Khayelitsha. The men were sampled using convenience sampling from Town Two, a neighbourhood in Khayelitsha, through a research assistant and community leader. Eligible men and women were approached at different times of the day at home, church, tavern and clinic and invited to participate in the study. The men were divided into two age categories: younger men (18–29 years) and older men (30–40 years) and discussions were held separately with each group on different days. The separation of young and old groups was to allow participants to freely express themselves [27].

One focus group discussion was held with 12 HIV-positive women, aged between 30–47 years. The women were selected purposively based on criteria of being HIV positive and were either currently accessing PMTCT services or had accessed PMTCT services not more than 2 years prior to this study.

In-depth interviews were also held with four couples with known HIV status. Key Informant interviews were held with five service providers including a nurse, midwife, doctor and two PMTCT counsellors, directly involved in PMTCT work at a clinic in Khayelitsha.

Key informant interviews with service providers were conducted and transcribed in English by the researcher and verified by two senior researchers as a form of quality check. Using semi-structured interview guide, responses were elicited from participants and tape recorded on a range of topics including: HIV testing, knowledge on MTCT, men`s role in PMTCT, experiences at antenatal clinics and infant feeding.

As this was an exploratory study of one community context, the purpose of the focus groups, couples interviews, and interviews with healthcare workers was to generate a broad description of men’s involvement in PMTCT and antenatal care, and explore what kinds of factors might enable or hinder that involvement (SI File). The number of focus groups and interviews were determined through saturation although the bar for saturation was set fairly low given the broad scope of the research objectives.

Focus group discussions were conducted in isiXhosa and transcribed verbatim into English. In-depth interviews were conducted in English and isiXhosa, transcribed verbatim by the researcher and a fieldworker, respectively. All interviews transcribed in English from isiXhosa were further verified by an experienced research assistant as a form of quality check.

Data was analysed using thematic analysis and summative content analysis [28, 29]. Transcripts were analysed using a four-step analysis as described in Dawson and Manderson, (1993). Individual transcripts were read and coded to reflect participant’s key responses. A log book was generated to group together similar/divergent topics from which themes were derived and further ideas for reflection and iterative analysis (S1 File). Results were written based on the log book and interpretation of the findings done in relation to study objectives and relevant contextual factors.

Emerging findings were reviewed by two senior researchers with extensive experience in HIV and PMTCT research in South Africa, and this particular field site. Data collected from men, women and key informants in the focus group discussions and individual and couple interviews were also triangulated to improve validity and reliability and refine the analysis. Access to data was restricted to only the researchers involved in the study and reference numbers were allocated to all FGD`s and interviews for confidentiality purposes.

Ethical approval for this study was obtained from the Research Ethics Committee of University of Cape Town and from the Provincial Department of Health of the Western Cape (REC REF: 142/2010). Participants provided written informed consent before the commencement of the study.

## Results

Table 1 describes the socio-demographic characteristics of participants in the focus group discussions. All men and women in the FGDs were Xhosa-speaking residents of Khayelitsha. Three women were pregnant at the time of the discussion, while two women had their babies present during the discussion. Demographic characteristics of the participants for in-depth interviews were as follows; Of the four couples interviewed, three couples were actively involved in a support group and employed as community volunteers, and were hence knowledgeable about HIV, PMTCT, and male involvement. Three couples were in a stable relationship and one couple was married.

**Table 1:** 

Socio-demographic characteristics of participants in the FGDs	Men	Men	Women
Age	18–29	30–40	30–47
No schooling		1	
Primary	4	5	2
Secondary	7	8	10
Employed		2	7
Unemployed	11	12	5
Single	9	7	7
Cohabiting	2	4	3
Married		3	2
Formula feeding			11
Exclusive breast feeding			1

The main findings from the study are presented and organized into three broad themes: 1) attitudes towards male involvement and PMTCT, 2) enablers, and 3) barriers to male involvement in PMTCT.

### 1) Attitudes towards male involvement in PMTCT

#### General attitudes towards male involvement

Most study participants were receptive towards pregnant women accessing PMTCT services like HIV testing, ART treatment, and using milk substitutes. While supportive of pregnant women accessing these services, however, most men perceived their role in PMTCT as consisting of financial and material support.

#### HIV-positive women becoming pregnant

A majority of men in the focus group discussions were, though, fairly strongly opposed to the general idea of HIV-positive women bearing children. Many men considered it unacceptable for HIV-positive persons to have biological children citing consequences such as placing a burden on social services and the family to care for the baby in the event of the parents’ death.

It is upsetting and unacceptable especially if a person knows that she has the virus and still becomes pregnant… **participant 12, older men’s focus group**.

A number of men in the older men’s group considered it a moral duty for HIV-positive women to give up their right to have biological children. Though many men thought it was a bad idea for HIV-positive women to become pregnant, they did not describe situations where this disapproval translated into neglect or punishment towards pregnant, HIV-positive women in their own lives.

There were some men in the younger men’s focus group who supported the idea of HIV-positive women having children on the condition that they sought medical care and followed clinical advice to prevent transmitting the infection to the babies.

#### Men’s and women’s HIV testing during pregnancy

All men interviewed in this study were in support of pregnant women testing for HIV during antenatal care visits.

… it is important that she gets checked so that the baby can be helped in time. **Participant 6, younger men’s focus group.**

The primary reason for supporting women’s testing was to safeguard the unborn baby from acquiring HIV. Women and men in the focus groups also expressed difficulties in initiating discussions around HIV testing and contraceptive use with partners.

… It was difficult to discuss HIV testing…asking her would have made her feel that she has been sleeping around with other men… It would make her feel that I don’t trust her… **male participant, accompanied partner for all ANC visits. Couple 4**

Many feared accusations of infidelity or lack of trust. Decisions on choosing or using a contraceptive method were entirely left to the women (except, for the use of condoms).

#### Men in antenatal care clinics

Most men interviewed in this study acknowledged that it was appropriate for men to accompany their partners to antenatal clinics.

That is the right thing to do, because going there, you are giving support to your partner who will be giving birth to your child and it is also right that when she gives birth you are here next to her… **Participant 9, older men’s focus group.**

Almost all male participants in this study supported the idea of men accompanying their partners for antenatal care with an exception of one participant in the young men’s focus group that disagreed with this notion due to stigma.

We all don’t see it as a good thing to do, because fun is being made by others when they see you with your partner (at the ANC clinic), worse we young people. **Participant 10, younger men’s focus group.**

All women interviewed expressed a desire for their partners to be present during antenatal sessions and delivery. Most women in this study had disclosed their status to partners or were single at the time of the discussions and likely to be receptive towards male involvement. There didn’t seem to be any feeling in the group of women that it was awkward or inappropriate for men to be involved with the medical care of their pregnant partners.

#### Infant feeding

All participants interviewed in this study were in support of and/or highly recommended formula feeding for infants whose mothers were HIV positive. There seemed to be general understanding that this helped to prevent one of the routes of vertical transmission of HIV. Some male participants argued that since formula milk is free in all public health facilities, all HIV-positive mothers should refrain from breastfeeding their babies to minimise risks of HIV infection. Women, on the other hand, felt that they should be able to decide what feeding option was suitable for them based on their own circumstances, especially their occupational requirements.

I told him it’s better if we do formula feeding and also breastfeeding would mean I would not have time for my classes.… so he said that if its okay with you, then its okay with me. **Discordant couple, female participant, actively involved in a teenage support group, Couple 3**

#### Acceptability of interventions and opportunities for engagement

Despite the fact that men in the focus groups all held generally positive views of PMTCT and partner support, they did convey a sense that the broader community of men was more diverse in their opinion.

That is the right thing to do, because going there is not something that you should be ashamed of and going there you are giving support to your partner who will be giving birth to your child and it is also right that when she gives birth you are here next to her and that is something not bad because that time when men were told they cannot get in those rooms are over and maybe that will also give her trust to see you there and make her strong. **Participant 9, younger men’s focus group.**

We all don’t see it as a good thing to do, because fun is being made by others when they see you with your partner, worse we young people. **Participant 10, older men’s focus group.**

In this study, men who were married or in a stable relationship and were living together with the partner were more prone to have been involved. Younger men and/or men who had ‘grown up’ in the urban areas were more open on participation in PMTCT. Older men and/or men with casual partners were more prone not to be involved. Age and marital status may be linked to men’s decision on whether to participate in PMTCT or not.

### 2) Enablers to male involvement in PMTCT

This study identified a range of enablers to male participation including: HIV testing, health worker contact, influence from peers within a support group, reaching out to men directly, knowledge and information flow on HIV/PMTCT, trust, and disclosure of HIV status.

#### HIV testing

Men who had tested for HIV and/or knew their sero status tended to be supportive of their partners throughout pregnancy and childbirth. Men who knew their status often accompanied their partners to antenatal care, encouraged their partners to test for HIV, and supported their partners in choosing an infant feeding option most suitable to their circumstances.

I was already aware (of my HIV status) and I had information about HIV/AIDS … It took time for me to convince her to go for a test, of which she did and she tested HIV positive and was also found to be pregnant … I knew that it was my chance to play my role as a man, to stand in my shoes, to show my love and support, so now she must not be afraid of anything… **male participant, tested before wife knew status, activist, Couple 1**

One man in the couple interviews claimed that the appreciation he received from nurses and explanations on how to support his wife while accessing couple testing had influenced his decision to be actively involved.

#### Role of support groups

Men who regularly attended some sort of support group gradually adopted the notion of accompanying their partners to clinics. Peers within a support group often encouraged and motivated each other to support their partners during pregnancy and child birth.

My friend told me to invite my partner to the support group and after the teenage (support) group, I went for a booking date, since then he has been caring for me during pregnancy and after pregnancy, he still cares for me and the baby. **Discordant couple, female participant, actively involved in a teenage support group, Couple 3**

Support groups also helped HIV-positive women cope with discrimination as they provided emotional support to each other. Women had stronger ties with members of the support group compared to family members.

#### Accommodative and flexible services

In this study, men were more willing to accompany their partners to distant clinics away from the communities in which they were known for fear of a breach in confidentiality from service providers.

Nurses that live closer in the area, she is the one that offered you help. There she goes and have tea with the neighbour about you, for example, you went for a test then the results came out the other way then she will go and tell the neighbour you are infected…but at least if it is far, even if she tells, I’m not known there… **participant 10, younger men’s focus group.**

#### Health worker contact

A majority of men expressed willingness to participate in PMTCT if they received information from health workers compared to receiving information from their partners. Information from service providers was considered more trustworthy and easily understood compared to what their partners told them.

It is very easy when something comes with the father or man especially if he understands it then brings it into the house … when this information [PMTCT] are said by women, its very hard for a man to understand it because they don’t give you detailed information about these things, they just force you in doing it… **participant 12, older men’s focus group.**

#### Prevention messages

An unexpected finding from this study highlighted the impact of prevention messages in the media that stigmatise and do not include men while relaying PMTCT information.

If posters could stop putting women only and start putting men who will say I am a man, I am HIV positive and I support PMTCT. That is where I think the men will be strong because they have seen from this guy that have disclosed his status and even those who were afraid maybe, they will come forward and talk about it. **Participant 9, women’s focus group.**

### 3) Barriers to male involvement in PMTCT

This study identified a number of barriers to male involvement including; stigma, negative attitudes from nurses, inadequate space, and staff shortages. Fear of breach of confidentiality, long waiting times, and being uncomfortable attending clinics where a majority of patients were women were also cited as reasons for low involvement in PMTCT services.

#### Men’s reluctance to engage in the clinic space

Men were reluctant to go to clinics that were considered to be ‘women’s spaces’. A number of men interviewed reported feeling uncomfortable attending a clinic with a majority of patients being women.

when sitting down on the chairs, you see women all around you and you end up shaking because you are asking yourself, are you sure of what you are doing here and you start to have second thoughts and the things they talk about are away from what men talk about. **Male participant, partner pregnant, couple 2.**

Almost all men interviewed in the couple interviews confirmed that it was difficult attending antenatal clinics for the first time, though with frequent visits, they became used to being around women.

What made me feel the pain was when people thought that I have bewitched this man, why he is here (ANC clinic). **Female participant, partner accompanied her for all ANC visits. Couple 4**

Some nurses had attitude towards me saying, you can see that it’s a clinic for women only and why can’t you wait outside for your partner. **Male participant, accompanied partner for all ANC visits. Couple 4**

Both men and women were, for example, were called names or accused of witchcraft while attending antenatal sessions with partners. Gender stereotypes still exist concerning men going to clinics ‘reserved’ for women discouraging some couples from attending antenatal care together.

A majority of women interviewed in this study reported discrimination during delivery and while using replacement feeding for the baby. Breast feeding was traditionally the preferred method of infant feeding in the community and formula feeding associated with being HIV-positive.

The nurse that will come in the place of the nurse that will be leaving for home, the one that is leaving will tell the one that is coming in that this one, and this and the other one are HIV positive, so they are being pointed out…. **Participant 6, women’s focus group.**

when they are sitting in the labour ward looking at each other and see those who give their babies breast milk and some would find that they don’t want to give their babies formula because they know that they will know she is positive. **Service provider 3, key informant interviews.**

The Department of Health dispensed one brand of formula milk to HIV-positive women in all public clinics in South Africa, known as Pelagon. This brand has come to be strongly associated with being HIV positive. Some women opted to‘re-label’ the formula milk received from clinics by using other milk tins other than Pelagon to avoid being labeled as HIV positive.

Stigma from both peers and health workers discouraged many men from going to test for HIV or attending antenatal clinics with partners, with the exception of one participant who saw the advantage of being helped outweighing the problem of his status being revealed. Some men opted instead to go to distant clinics were they were not known with partners.

In-depth interviews with service providers confirmed that clinics did not have the capacity to accommodate all male partners of pregnant women accessing antenatal care due to inadequate space.

We do not have space to accommodate every body`s partner…maybe they (men) can come to help if she has given birth to twins. **Service provider 1, key informant interviews.**

Some men in the focus group discussions highlighted long waiting times at the clinic as a deterrent to them accessing services as well. Staff shortages were an issue since involving men required more consulting time. Men were encouraged to come in only during labour or when a partner has had multiple births due to the perceived additional help needed by the mother.

#### Non-disclosure

Non-disclosure of HIV-positive status by women was a barrier to male involvement. Discussions with service providers and women revealed that disclosure often served as an entry point to partner involvement during pregnancy.

…it all depends on that person. Does she want to disclose, does she want to be known that she is HIV positive. So you get that problem that they don’t disclose … then it’s gonna be a problem to say to the man to come (to the clinic). **Service provider 2, key informant interviews**.

However, some women in the focus group discussion had problems disclosing their HIV sero-positive status to partners citing fear of abandonment, rejection, and violence as reasons for non-disclosure.

The minute you tell them, they pretend to have accepted it, then telling themselves inside that “I will beat you up and ask you where you got this virus whereby I don’t have it”. How will he know if he did not go and get tested.… then he will go to work and never return… **Participant 10, women’s focus group.**

A perceived fear of being blamed for the disease prevented several women from disclosing their status to partners. Some women gave excuses when asked about the medication (Nevirapine) they were taking during pregnancy, explaining it was ‘for the baby’s growth’. Mixed feeding was reported to be common in women that had not disclosed their status.

## Discussion

This study showed that both community members`(men, women) and health care providers were receptive of male involvement in PMTCT services. However, there were differences in perceptions of what constituted male involvement amongst men, women and health care providers.

Direct health worker engagement with men, disclosure of status by women and HIV testing, enhances increased male participation in maternal and child health. This finding is similar to a qualitative study conducted in Vietnam that showed that men who had tested for HIV and/or disclosed their HIV status were more supportive of partners throughout pregnancy and childbirth [30]. Men’s participation could also be shaped by the role of health workers who provide information on HIV/PMTCT and describe men’s roles in family care in ways that extend beyond meeting material needs to supporting the pregnant partner in monitoring ART compliance, infant feeding compliance, and emotional support. The role of health workers in persuading men`s uptake of PMTCT services has been documented elsewhere [31].

Men who regularly attended some sort of support group were more likely to attend antenatal clinics with partners. Peers within a support group often encouraged and motivated each other to support their partners during pregnancy and child birth. The support group’s women attend such as those run by the NGO mother2mothers, create a bond among the pregnant women accessing PMTCT. Support groups are like family and often they refer to each other as ‘comrades’ providing support to members. The role of peer to peer support has been documented in studies conducted in Uganda and Zambia [32, 33].

HIV-positive women also had weak social ties with family members but stronger social ties with members of the support group they belonged to. This finding is similar to a study conducted among HIV-positive women and men in Soweto, South Africa that showed that HIV placed a tremendous strain on familial relationships [34]. Overall, men were knowledgeable about PMTCT and the potential roles they could play in the programme. A discrepancy exists in this study, however, between what men know and consider as their roles in PMTCT and what their actions concerning their involvement depict. This discrepancy was elucidated in a study conducted in rural Uganda that cited reasons for men`s non-participation in HIV testing despite being knowledgeable such as mistrust in marriages, rude health workers, lack of motivation and convincing messages to warrant their involvement [32,35]. Men’s roles in PMTCT need to be clearly defined and appropriate programs expanded to reach out to men in all spheres of life to embrace these new roles in family care.

The findings suggest three kinds of men; men who are or would be actively involved in PMTCT, men who are not involved but could be motivated, and men who are resistant to getting involved in any kind of support for their partners (except perhaps for financial support). The men who are involved in PMTCT are more likely to have tested for HIV, are knowledgeable about HIV/AIDS, and are generally positive about their involvement in the partner’s pregnancy. Men who are not involved and uninterested were more likely to resist intervention messages and/or discussing reproductive health, HIV/AIDS or PMTCT with their partners. However, for the men in the middle, they may be less concerned about or aware of PMTCT but may not necessarily resist intervention messages. There is a possibility of them being actively involved if reached out to appropriately. Social relationships of care and decision-making are changing and men are finding themselves in situations where they are being asked to do more in the family than before [36]. The demands on men to be actively involved in family care are increasing in light of urbanization, gender equality movements, and the HIV/AIDS epidemic in South Africa [19]. Men’s roles in Africa are increasingly extending beyond the traditional roles of being the ‘bread winner’ in the family to attending antenatal care visits, making birth plans, caring for babies and monitoring ART and infant feeding options for the mother and the baby [15,20,21]. Older men in this study resisted these new care-giving roles more strongly than younger men claiming it would make them lose respect from their peers. In contrast, Ditekemena et al reported that men who were older and in monogamous relationships were more likely to embrace the care-giving roles accrued to them [37].

## Conclusion

Support groups and direct health worker engagement with men increases male involvement in PMTCT services. Using men in the media and community to reach out to fellow men with prevention messages tailored to suit specific audiences may reduce perceptions of antenatal care as being a woman`s domain. Promote the benefits of male participation in the media using couples as flag bearers of PMTCT information to encourage family-centred care.

## Limitations of the study

This study explored perceptions of community members’ (men and women) and healthcare workers on male involvement in ANC and PMTCT services that provided an opportunity to compare different views and opinions from participants. The views and opinions expressed in this study may not be generalised nationally, although useful insights can be drawn from it to improve existing PMTCT interventions. The study used a Xhosa-speaking facilitator enabling participants to express themselves candidly though translating the interviews into English may have affected the originality of the field data collected and limited the researcher`s ability to probe.

## Supporting Information

S1 File (DOC)
