# Supplementary material for: Perceptions of Community Members and Healthcare Workers on Male Involvement in Prevention of Mother-To-Child Transmission Services in Khayelitsha, Cape Town, South Africa
Source: PLoS One. 2015 Jul 28;10(7):e0133239. doi: 10.1371/journal.pone.0133239 (PMC4517758; doi:10.1371/journal.pone.0133239)
Supplement: S1 File — (DOC) [file pone.0133239.s001.doc]

**Themes from raw data**

**Men**

Knowledge/understanding on PMTCT

What men thought women`s expectations of their roles in maternal and child health were

Child bearing in HIV positive women

Gender roles and norms – access to information, care-giving roles

Men`s perceptions of their role in HIV prevention in infants

Infant feeding in HIV positive women

HIV testing – communication/initiation

Challenges in attending ANC

**Women**

Challenges in partner involvement in ANC

Men`s role in pregnancy and child birth– ANC and Post natal care

Choices on infant feeding

What aspects of support did they want from their partners

Gender norms around access to PMTCT

Experiences with health care workers

**Extracts**

**Focus groups**

**Men**

| **Themes/topics** | **Extracts** |
| --- | --- |
| Most participants were aware about PMTCT, though were not so sure of what happens beyond pregnant women accessing VCT and neverapine. |  |
| How men show support to pregnant partners   - Financially - Emotional –love and care - Physically – helping out in house chores   N.B more like what men should do when the partner is pregnant… expectations from women/obliged to do? |  |
| Men`s role in PMTCT  -condom use  -faithfulness to intimate partner  -HIV testing  avoid substance abuse  accompany partner to the clinic, ensure she`s taken her medication, help with the chores at home, buy food. |  |
| All agreed on the importance of both partners being given PMTCT information.  However the information should start with the men first…  -culture  -gender norms and social upbringing | “…they should have started with the fathers being the head of the house…it’s very easy when something comes with the father or man especially if he understands it then brings it to the house… when it comes with the mother, it’s not very easy for me to understand…” P.12.  “…men should be engaged more…I don’t want to lie it’s hard for us men. Even when told it goes from this ear to another ear…who am I to use a condoms or have a single partner…whereby my father maybe had too many partners and that`s the problem” P.9. |
| Infant feeding in HIV positive women  All agreed formula milk was the best to avoid mother to child transmission. |  |
| Challenges in attending ANC  Negative attitude from nurses  Staff shortages, Long waiting times and being sent away  Fear of stigma and a breach in confidentiality  Prefer to go for HIV testing were they are not known. | “…the problem is that when you go to a clinic that is near, the people from here, they have a big mouth.” P.3  “…nurses that live closer in the area, she is the one that offered you help, there she goes and have tea with the neighbour about you, for example, you went for a test then the results came out the other way then she will go and tell the neighbour you are infected” P.5 |
| Men accompanying their partners to ANC clinics  Majority found it acceptable for men to accompany their partners to ANC clinics.  A few disagreed because – of stigma and some ridicule from peers. (Young people). | “we see it as the right thing and its educating to other people, let’s say you were afraid that people are going to make fun of you, but when you see it being done by someone else you then end up finding it as the right thing to do when taking your partner to the clinic” P.4  “I also see it as the right thing to do because in the past times, these things were not allowed, so if you were to go to the clinic it’s the right thing and sometimes you would find that maybe your partner went to the clinic and came back not feeling well, maybe she got a miscarriage then you get angry whereby if you were with her or had information then you would understand what happened to her” P.8 |
|  |  |
|  |  |

Women

| Themes /comments | Quotation |
| --- | --- |
| Men`s role in pregnancy and child birth– ANC and Post natal care  How men offer support | “...pregnancy is not something that just happens, you plan to get pregnant...when you want to have a baby, they should sit down and talk, check their HIV status so they can keep using condoms...he must sacrifice his working time so that he can go with you, meaning that it would be very nice to see him next to you...” P.10  “men should play the role of doing some house chores like washing and cleaning...you would find that there is only one nurse at the MOU who is busy attending to other patient...and you are having pains...the men will help you by maybe brushing your stomach in the meantime...” P.9  “...during your time of pregnancy its worse in the 1^st^ trimester there is a lot of fighting that happens in the house, men become abusive so at least if men could understand when his partner is pregnant what must he do, must he wash your feet, do some massage...those are the things men don’t do and must do to show some responsibilities, so that is why you find a lot of fighting happening...” P.8  “...sometimes you go to the labour ward in pains already and then if your partner knows your status then he`s able to disclose to the nurses so that AZT and NVP is given...” p.6  “...that time when im choosing the method to feed the baby, he should be next to me so that he can know what is happening and when this milk will be finished and not accept this milk will take a year, so that he knows that milk is running out and try and avoid fights taking place at home” P.10 |
| Challenges of male involvement  Lack of information  culture | “… Other men when they find that you are pregnant and found to be positive then you tell him he will tell you it’s only you that has this I’m not positive and that is why you would find that some women don’t disclose to their partners, others give breast milk to their babies even if they know that they were not allowed to give their babies breast milk, because they are afraid of braking up with the father of the child.” P.1  “...you are infected with HIV he denies that he is also positive, then he will go to work and never return again then you start to have depression because you have the baby that you have to look after then he has left then you are HIV positive so as many have said you will have depression, so since this culture thing if a women knows her status first it’s hard for her to tell but if a man tells or gets sick first it’s hard for the women to leave that man alone because she has to look after him and put her nails in taking care of these men, but if she tells him her status he runs away and never come back again...” P.7 |
|  | “...the minute that they find out that you are HIV they leave you’re unattended, up until some people give birth for themselves. Our people come back complaining about the nurses.” P10  “I have to agree she is right if you take someone there, the nurse that will come in the place of the nurse that will be leave home, the one that is leaving home will tell the one that is coming in that this one, and this and the other one are HIV positive, so they are being pointed out and they tell the other nurse that entered releasing the home”. P.6  “...you are told that your date of delivery is still too far from giving birth or the water in the womb have not yet bust, so other people tell that they have been in pains since last night or they tell you that don’t be in a hurry go home or others maybe approaching the gate then gives birth after being told to go home.” P3. |
|  | “…Other thing that I did not mention is that women should not be the only people that disclose their status and if the posters could stop putting women only and start putting men who will say I am a man and I got my girlfriend/wife pregnant and I am HIV positive and I support PTMCT and that is where I think the men will be strong because they have seen from these guy that have disclosed his status and even those who were afraid maybe they will come forward and talk about it.” P.9 |
|  | “… Other thing that makes it difficult to discuss and disclose is that men run away. Once you tell a person that you are living with HIV a person decides to run away...”P.4  “...the minute you tell them they pretend to have accept it then telling themselves inside that I will beat this one up and use his strength to beat you up and ask you where you got this virus whereby I don’t have it, how will he know if he did not go and get tested. How will he know if he if he has it or not. “P.8.  “Men like to make themselves stubborn about the things that they know, he will go and bring you HIV and if you tell him about the HIV makes like he never heard about it before. “ P.13  P.3 ” ...when you ask them about these things they become very angry and it breaks everything that you guys had because of the anger that he has about HIV.”  “...that you are infected with HIV he denies that he is also positive, then he will go to work and never return again then you start to have depression because you have the baby that you have to look after then he has left then you are HIV positive... this culture thing if a women knows her status first it’s hard for her to tell but if a man tells or gets sick first it’s hard for the women to leave that man alone because she has to look after him and put her nails in taking care of these men, but if she tells him her status he runs away and never come back again.” P.7 |
| Reasons for non disclosure  Stigma, partner denial, fear of break up /rejection, violence |  |

**In –depth interviews**

Couples

Couple one.

| Themes/comments | Quotations |
| --- | --- |
| Male partner | “...It was my chance to play my role, to stand in my shoes, to show my love, my care and support. So now she must not be afraid of anything about me, she must trust me because I love her...” (discordant HIV results – man tested negative and woman tested positive at ANC).  “You see the mother was confused when she found out that she was positive and pregnant ...she had to tell me of which she was very confused on how she will put it to me....every day I made sure that she was healthy and made sure that the baby would be secured (PMTCT program) I also gave her alot of books to read about HIV...”  “...It was difficult to discuss HIV testing...asking her would have made her feel that she has been sleeping around with other men... It would make her feel that I don’t trust her...” |

Couple .two

| Themes /comments | Quotation |
| --- | --- |
| Male partner | “...some understand and those are the ones who you have met already and some that have strange faces and give you names saying you are homosexual or you are a cross gender...”  “...when sitting down on the chairs, you see women all around you and you end up shaking because you are asking yourself, are you sure of what you are doing here and you start to have second thoughts and the things they talk about are away from what men talk about...”  “...if I was not there, then I will have too much questions but once I go there, then I will see why she wanted me to go with her...what you are supposed to do when the baby is born, what you must support her from the start not support her financially only but emotional and physically right through the end so that you have created a bond with your baby...so that is what occurred to me when she asked me...” (to accompany her to ANC) HIV positive couple, with an HIV negative baby, chose formula feeding for the infant. |

Couple three

| Themes /comments | Quotation |
| --- | --- |
| Male partner | C.2.M  “I don’t want to lie, all I heard was compliments, saying that it is very rare for men to come to the clinic with their partners and encouraging the ladies to also bring their men along with them to the clinic and some said she (partner) was lucky that her man came to the clinic with her.” |
| Female partner | “... my friend told me that there is a support group for teenage mothers and told me that I could bring my partner and maybe he might be able to help me and after the teenage group, I went for a booking date, since then he has been caring for me during pregnancy and after delivery, he still cares for me.”  “what made me feel the pain was when people thought that I have bewitched this man, why he is here(ANC clinic) and some of the nurses told me that I`m so lucky because African men don’t want anything to do with the clinic...” (reinforcing gender norms)  “ we discussed them (infant feeding options) when I was 8 months pregnant...I told him its better if we do formula feeding and also breast feeding would mean I should be with my child all the time and I would not have time for my classes...so he told me that if its okay with you, then its okay with me” |

**Health care workers**

Benefits of male involvement in PMTCT

Challenges of involving men in ANC

HCW role in enhancing male involvement

Men`s role in PMTCT.

Contraception use

Infant feeding

HIV testing and disclosure

**Key Informant Interviews**

| **Participant** | **Themes** | **Quotation** |
| --- | --- | --- |
| Key Informant.1. (K.I.1) | **Benefits of male involvement**  Adherence /compliance   1. ART 2. Infant feeding option | `if the partner is involved, then it is easy for the mother to care for the child appropriately, in the sense that, if she chooses to formula feed or breast milk, the partner will have the knowledge to prepare the milk or know that its nothing else but the breast milk.`  “ it is easy for the mother to comply with whatever choice of baby feeding or medication if the partner has been informed”. |
|  | Role of HCW in enhancing increased male involvement | “...the partners are encouraged to come and get involved when the woman is starting medication, to help in monitoring compliance”  “...educating the partner is very beneficial for the mother..  “We have a support group and a peer counsellor for the mothers that are breast feeding. So if the men can come and be educated with their partners” |
|  | Challenges of getting men involved  Space  Culture  -gender roles  Changing patterns in men`s perceived roles/masculinities.  Time constraints | “we do not have space to accommodate every body`s partner...but we are not against anybody who comes with their partner..”  “culture also plays a role in the men not coming to the labour ward, saying its a woman`s thing but with the westernization, some do come and we let them in.  “most of them do work and may not be available to come with their partners” |
|  | Role of HCW in enhancing partner involvement in ANC and PMTCT. | “when the women come to book, we do encourage them to come with their partners and access HIV testing together and facilitate disclosure” |
|  | Emotional support | “when the mothers come and have been newly diagnosed with HIV and still in shock, they go to the support group and their anxieties are relayed” |
| Key Informant.2. (K.I.2) |  | “ I think both partners, because the problem we are getting with the women is that they don’t comply, they only get the treatment and they will not come back. but if given to both, then the other one can ask or can give that support to the other partner, like the male can give a female the support of taking the treatment to prevent the baby to be infected”  “but the problem with them (women) is that they do not disclose –either they are scared that the boyfriend or husband is going to leave them”  “you get that problem that they don’t disclose. So it will be difficult if they don’t disclose. It is much easy if they disclose, then the partner can know and give that woman the support”  “ you can just advice that it is much better if your partner know that you are HIV positive, lets say you cannot come and take your treatment, he can come and get it for you”  “we do advice them that since they are HIV positive and have got two children already, to use contraception because of the risks involved...you hear them when they are talking with their peers in the corridors that the reason why they are falling pregnant each and every time is because of the grant money. You find that if she`s got 4 children, each child is given 250 rands per month...it is a source of income for the mother who is not working...”  “...we do try our best to give them information on family planning. They only get a contraception method after delivery. When you give them the appointment card for the next appointment, they do not go. You only find out that this woman was pregnant last year and she is back again...”  “there are cases when the women want to disclose to their partners but the are scared. Women are then encouraged to bring their partners to the health facility to facilitate disclosure with a counsellor`s help...” |
| Key Informant.3. (K.I.3) | Peer influence  Fear of stigma | “when they are sitting in the labour ward looking at each other and see those who give their babies breast milk and some would find that they don’t want to give their babies formula because they know that they will know she is positive”  “People don’t want to use contraception, they say injections block them. I don’t have period pains when I’m using the injection” |
| Key Informant.4. (K.I.4) |  | “I mean as a HCW, I don’t think you can play a big role in that it all depends on that person. Does she want to disclose? Does she want to be known that she is HIV positive? Other people have got stigma towards other people that have tested positive...” |
